# Supplementary material for: A Machine Learning Approach Unveils the Relationships between Sickness Behavior and Interoception after Vaccination: Suggestions for Psychometric Indices of Higher Vulnerability
Source: Healthcare (Basel). 2023 Nov 18;11(22):2981. doi: 10.3390/healthcare11222981 (PMC10671008; doi:10.3390/healthcare11222981)
Supplement: Supplementary file 1 [file healthcare-11-02981-s001.zip › healthcare-2657463-supplementary.pdf]

Supplementary Material

1. Table S1: first section of the online survey.
2. Table S2: trait characteristics.
3. Supplementary File S1
4. Table S3: confusion matrix referring to the first preliminary round.
5. Table S4: common classifiers referring to the first preliminary round.
6. Table S5: confusion matrix referring to the second round.
7. Table S6: common classifiers referring to the second round.
8. Table S7: confusion matrix referring to the last round.
9. Figure S1: SicknessQ Boxplots.

Table S1. First section of the online survey

Questions	Possible answers
Sex	· Male<br>· Female<br>· Other
Age	Free answer
Marital Status	· Unmarried/maiden<br>· Married<br>· Separated<br>· Widower<br>· Other
Level of education	· Primary school<br>· Secondary school<br>· High school<br>· Bachelor's degree<br>· Master's degree<br>· PhD<br>· Other
What is your profession? (If you have difficulty identifying your sector, consult this site: https://www.atlantedelleprofessioni.it/professioni)	Free answer
Do you usually practice physical activity?	· No<br>· Yes, less than once a week<br>· Yes, once or twice a week<br>· Yes, three or more times a week<br>· Other
If you do physical activity, can you tell us what kind of activity?	· None<br>· Fitness<br>· Running<br>· Swimming<br>· Walk<br>· Football<br>· Other
Have you ever been affected by COVID-19?	· I've never been positive about Covid<br>· I am currently positive for Covid<br>· I had Covid but I am no longer positive (swab result: negative)<br>· Other
Has the doctor ever diagnosed you with major diseases? (If yes, please specify)	Free answer
Were you in an immunocompromised situation during vaccination (e. g. immunosuppressive therapy, immunodeficient disease)?	· Yes<br>· Not
Did you experience any side effects from the vaccination?	· Yes<br>· Not
Questions	Possible answers

If so, which ones?	· No reaction<br>· Tenderness, pain, warmth, itching or bruising where the injection is given<br>· Feeling tired (fatigue) or generally feeling unwell<br>· Chills or a feeling of fever<br>· Headache<br>· Feeling sick (nausea)<br>· Joint pain or muscle pain<br>· Swelling or redness where the injection is given<br>· Fever (> 38 ° C)<br>· Malaise (vomiting or diarrhea)<br>· Drowsiness or feelings of dizziness<br>· Decreased appetite<br>· Enlarged lymph nodes<br>· Excessive sweating, itching or rash<br>· Other
How worried were you about possible adverse reactions?	The answer uses a ten-point Likert scale ranging from 1–10 (from “not at all” to “very much”)
Did you hear about adverse vaccine reactions prior to vaccination?	· No<br>· Yes, from the TV<br>· Yes, from social media<br>· Yes, from friends or relatives<br>· Yes, from colleagues<br>· Yes, I had talked to my doctor about it<br>· Other
If you answered “Yes” to any of the above options, did you take any precautions?	· Yes<br>· Not
If so, what precautions?	Free answer

Table S2: trait characteristics		
**MAIA components**	Median	Interquartile Range
**Noticing**	2.25	[1.00 – 3.00]
**Not-Distracting**	2.33	[1.66 – 3.00]
**Not-Worrying**	3.00	[2.33 – 3.66]
**Attention Regulation**	2.42	[1.60 – 3.28]
**Emotional Awareness**	2.80	[1.00 – 3.60]
**Self-Regulation**	1.80	[1.00 – 2.60]
**Body Listening**	2.00	[1.00 – 3.00]
**Trusting**	3.00	[2.00 – 4.00]
**Concern about adverse reaction**	4.00	[2.00 – 6.00]

Supplementary File S1

Classification accuracy between L-SB and H-SB groups based on all variables, including SicknessQ, at the baseline

The baseline variables exhibiting optimal efficiency in classifying the participant within the L-SB and H-SB groups have been identified through JRIP rule (Cohen, 1995 [18]) and its cut-offs. JRIP yielded an accuracy of **76.20 %**, as shown in the decision matrix (**Table S3)** and the rules (n. 6) were the following:

1) If SicknessQ Total Score T0 is ≥ 3, then the subject is classified as H-SB;

2) If SicknessQ Total Score T0 is ≥ 1 and MAIA Emotional Awareness is ≥ 3.6, then the subject is classified as H-SB;

3) If Systemic Symptoms = Positive and Sex = Female and Age ≤ 53 and MAIA Noticing ≥ 1.25 and MAIA Not-Worrying ≥ 3, then the subject is classified as H-SB;

4) If Systemic Symptoms = Positive and PSQI Subjective quality of sleep *T0 ≥ 1 and Concerns about adverse reactions ≥ 6 and PSQI Sleep Latency T0 ≥ 1, then the subject is classified as H-SB;*

5) If Fever (> 38 ° C)= Positive and Local symptoms= Positive and Concerns about adverse reactions ≥ 4 and MAIA Attention Regulation ≤ 2.714286, then the subject is classified as H-SB;

6) If the previous five roles are not applicable, then the individual is classified as a L-SB subject.

The reported set of decision rules results in high accuracy in classifying High-SB (176/227; accuracy=78%) and Low-SB (189/252; accuracy=75%) groups, AUC= 0.710 (d= 0.782) and F1=0.76.

Besides *JRIP,* the classification has been performed also by five other common classifiers: Naïve Bayes, Logistic Regression, Simple Logistic, Support Vector Machine, Random Forest. Their classification accuracy was estimated using k-fold cross validation technique (k=10) yielding performance similar to JRIP (**Table S4)**.

In particular, the most accurate classifier in classifying individuals with low SB (209/252) and high SB (174/227) (83% and 77%, respectively) was the Support Vector Machine (SVM) with an overall accuracy of **79.96**%, AUC =0.729 (d=0.862). However, compared to JRIP the other classifiers did not allow identifying variables exhibiting optimal classification efficiency.

Table S3. Confusion matrix referring to the first preliminary round.

	L-SB (Group A) (n=252)	H-SB (Group B) (n=227)	Accuracy for corrected classification
L-SB	189	63	75%
H-SB	51	176	78%

Confusion matrix showing the clearer performing set of decision rules (n=6) with JRIP using 10-fold cross-validation (AUC=0.710, d=0.782; F1=0.76)

Table S4. Common classifiers referring to the first preliminary round.

Classifier	Accuracy (%)	AUC	F1	Correct classification
Naïve Bayes	77.04	0.835 (d=1.378)	0.76	L-SB: 229/252; H-SB: 140/227
Logistic Regression	77.04	0.827 (d=1.333)	0.77	L-SB: 198/252; H-SB: 171/227
Simple Logistic	78.50	0.833 (d=1.366)	0.78	L-SB: 211/252; H-SB: 165/227
Support Vector Machine	79.96	0.729 (d=0.862)	0.80	L-SB: 209/252; H-SB: 174/227
Random Forest	79.54	0.848 (d=1.454)	0.80	L-SB: 209/252; H-SB: 172/227

The correct classification achieved by the different classifiers measured by accuracy, AUC, F1.

Table S5. Confusion matrix referring to the second round

	L-SB (Group A) (n=252)	H-SB Group B) (n=227)	Accuracy for corrected classification
L-SB	171	81	68%
H-SB	50	177	78%

Confusion matrix showing the clearer performing set of decision rules (n=8) with JRIP using 10-fold cross-validation (AUC=0.692, d=0.709; F1=0.726)

Table S6: Common classifiers referring to the second round.

Classifier	Accuracy (%)	AUC	F1	Correct classification
Naïve Bayes	75.16	0.817 (d=1.278)	0.75	L-SB: 222/252; H-SB: 138/227
Logistic Regression	75.99	0.794 (d=1.160)	0.76	L-SB: 197/252; H-SB: 167/227
Simple Logistic	78.08	0.819 (d=1.289)	0.78	L-SB:199/252; H-SB: 175/227
Support Vector Machine	77.04	0.706 (d=0.766)	0.77	L-SB: 195/252; H-SB: 174/227
Random Forest	77.24	0.822 (d=1.305)	0.77	L-SB:197/252; H-SB: 173/227

The correct classification achieved by different classifiers measured by accuracy, AUC, F1

Table S7: Confusion matrix referring to the last round.

	L-SB (Group A) (n=252)	H-SB (Group B) (n=227)	Accuracy for corrected classification
L-SB	181	71	72%
H-SB	47	180	79%

Confusion matrix based on the JRIP set of decision rules, using 10-flod cross-validation. (AUC= 0.694; d=0.717; F1= 0.754)

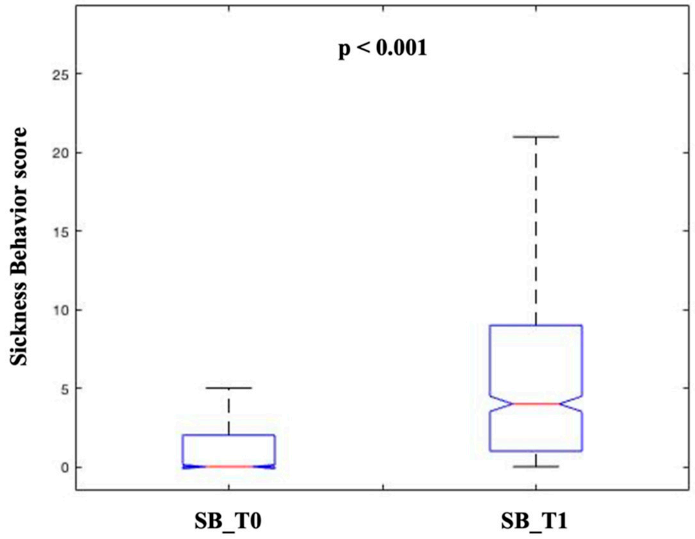

Figure S1. Boxplots show the increase in SB levels before (SB_T0) and after (SB_T1) vaccine inoculation. Boxes represent the first and third quartiles, and horizontal lines within boxes represent median values. Whiskers represent non-outlier high and low values.
